# The Biotransformation and Influence on the Functional Activities of Metabolites during the Fermentation of *Elaeagnus moorcroftii* Wall.*ex Schlecht.* Juice by *Bifidobacterium animalis* subsp.* lactis* HN-3

**DOI:** 10.3390/foods13060926

**Published:** 2024-03-19

**Authors:** Yixuan Wang, Chenxi Wang, Zhenghui Lan, Yingdi Teng, Yongqing Ni, Yan Zhang

**Affiliations:** 1 School of Food Science and Technology, Shihezi University, Road Beisi, Shihezi 832003, China; wangyixuan724@163.com (Y.W.); w835951299@163.com (C.W.); lanzhenghui0913@163.com (Z.L.); 19826099205@163.com (Y.T.); 2 Key Laboratory of Agricultural Product Processing and Quality Control of Specialty (Co-Construction by Ministry and Province), School of Food Science and Technology, Shihezi University, Shihezi 832000, China; 3 Key Laboratory for Food Nutrition and Safety Control of Xinjiang Production and Construction Corps, School of Food Science and Technology, Shihezi University, Shihezi 832000, China

**Keywords:** *Elaeagnus moorcroftii* Wall.*ex Schlecht.*, non-targeted metabolomics, antioxidant activity, microbial biotransformation

## Abstract

*Elaeagnus moorcroftii* Wall.*ex Schlecht.* (EWS) has extensive nutrients and functional active ingredients, which makes it an excellent potential substrate for fermentation. The improvement in the antioxidant activity of *Elaeagnus moorcroftii* Wall.*ex Schlecht.* juice (EWSJ) fermented by *Bifidobacterium animalis* subsp.* lactis* HN-3 (B.an3) could be attributed to the metabolism and biotransformation of plant-based products by the bacterial strain. To reveal the underlying mechanism, non-targeted metabolomics was applied in this study. After fermentation, the structure of downregulated carbohydrates, amino acids, fatty acids, and flavonoids was changed by *Bifidobacterium* biotransformation (included four reductions, three hydrolyses, four isomerizations, three deglycosidations, and five other reactions). The structure of these converted upregulated products has a higher antioxidant ability to reduce free radicals than their precursors, such as the flavonoids in the form of hydrolyzed conjugates, amino acids with multiple sulfhydryls or hydroxys, carbohydrates with reactive oxygen on benzene rings and fatty acids with unsaturated bonds, short chains, and glycosides. These findings shed light on the mechanism of the metabolism and biotransformation of EWSJ by B.an3, facilitate the study of the interaction between probiotics and fermented plant-based products, and provide a theoretical basis for the development of *Bifidobacterium*-fermented plant products with stronger functional activities.

## 1. Introduction

In recent years, the market for probiotic-fermented foods for the development of functional foods has immensely increased [1]. This trend has arisen because probiotic dairy products are not suitable for certain consumer groups with lactose intolerance or other illnesses resulting from the consumption of these products and for those adopting vegetarianism or veganism [2]. *Elaeagnus moorcroftii* Wall.*ex Schlecht.* (EWS) (Family: Elaeagnaceae) is a deciduous tree widely distributed throughout the western regions of China [3]. The fruits of the Elaeagnaceae species exhibit remarkable biological activities and are widely used to treat several health issues such as aging, burns, dyspepsia, diarrhea, pain, bronchitis, and neurasthenia [3]. In addition, various nutrients (e.g., sugars, proteins, fats, vitamins, minerals, and amino acids) and functionally active substances (e.g., proanthocyanidins, phenolic acids, tannins, flavonoids, terpenoids, and alkaloids) have been detected in the fruits of EWS [4]. These substances can be used as nutrients for probiotic fermentation and can enhance the functional activity of the probiotic [1]. In previous studies, the EWS juice (EWSJ) has been reported to contain many prebiotics, such as raffinose, which promote the growth of *Bifidobacteria* [1,4]. Furthermore, fatty acids occur naturally in the fruits of the Elaeagnaceae species and are the primary metabolite of anaerobic *Bifidobacteria* fermentation via the “bifid shunt” catabolic pathway [5]. In previous studies, the existence of the structure–activity relationship between functionally active substances and their structures has been reported [6,7,8,9]. Flavonoids occurring in the form of glycosides and methoxy conjugates possess lower antioxidant activity than their hydrolyzed conjugate counterparts [6]. Amino acids have multiple sulfhydryl or hydroxy groups, carbohydrates have more numbers of reactive oxygen molecules on benzene rings, and saturated fatty acids are transformed into unsaturated, short-chain fatty acids and fatty acyl glycosides, which increases their functionalities [7,8,9]. Therefore, the modification of the structure of functional active substances in a juice can further lay the foundation for the development of functional juice.

Microbial fermentation is an effective approach to induce structural modification through enzymatic reactions produced by microorganisms. *Bifidobacterium* has a large enzyme system, which acts on specific substances to biotransform their structures, such as through the production of hydrolase, oxidoreductase, ligases, isomerases, and transferases [5]. Meanwhile, *Bifidobacteria* are gram-positive polymorphic rods that are commonly used as probiotics for human consumption as they exert numerous positive effects on human health owing to their antibacterial, immunostimulatory, and anticarcinogenic properties [5]. After fermentation, probiotics not only promote their growth using the nutrients (e.g., prebiotics) in the plant-derived foods but also lead to an increase in the antioxidant activity (e.g., ABTS, DPPH, FRAP, and ORAC) and cause considerable changes in the functional substances (e.g., carbohydrates, amino acids, fatty acids, terpenoids, and flavonoids) of plant-derived foods [1]. Apple juice exhibited strong malolactic conversion ability and enhanced DPPH and FRAP antioxidant activities but decreased the total phenol and flavonoid levels during the fermentation of *Bifidobacterium lactis* 80 [10]. The antioxidant capacity was considerably enhanced in soy drinks after fermentation by *Lactobacillus paracasei* ssp. *paracasei* 431 and *Bifidobacterium animalis* ssp. *lactis* Bb-12 [11]. Nevertheless, the mechanism by which probiotics cause the metabolic transformation of different classes of substances and their specific functional activities is less often reported. For example, Sun et al. reported that the identified differential metabolites (e.g., organic acids, amino acids, and fatty acids) were linked to the tricarboxylic acid cycle, glutamate metabolism, and fatty acid metabolism in the probiotic-fermented milk during fermentation by *Bifidobacterium* and *Lactobacillus* [12]. However, the metabolic mechanisms have been chiefly investigated in dairy products, while the metabolic mechanisms of probiotic plant-based fermentation were unclear.

Therefore, we suspected that *Bifidobacterium* species have the propensity to promote the reductions, hydrolysis, isomerizations, and deglycosidations of functionally active metabolites in EWS juice during fermentation via the action of specific bifidobacterial enzymes, which then facilitate the identification of more active ingredients and increase the bioavailability of these substances in plant-based food owing to the bioactive structure. To verify our conjecture, this study aimed to reveal the mechanism of improvement in the antioxidant activity of EWSJ fermented using *B. animalis* subsp.* lactis* HN-3 (B.an3). This strain was selected for the fermentation of EWSJ to determine its effect on growth, pH, total soluble solids, total flavonoids, and antioxidant activity. With non-targeted metabolomic analysis, the association between metabolite transformation and the functional activity of metabolites in EWSJ using B.an3 fermentation was investigated. Furthermore, the regulation of metabolites, metabolic pathways, and mechanisms of biotransformation induced by B.an3 was summarized. Overall, the findings provided detailed information on probiotic metabolism in EWSJ and the potentially beneficial mechanism of probiotic-fermented plant-based foods.

## 2. Materials and Methods

### 2.1. Plant Materials and Bacterial Strains

#### 2.1.1. Preparation of EWSJ

The red or yellow, wild, and ovular fruits of EWS (15–25 mm in length) were picked from fully mature plants grown in natural dry saline soil in Kashgar in Southern Xinjiang Province in China (73°20–79°57 N, 35°20–40°18 E). The sugar, acid, and protein content of EWS fruits were 415–645 mg/kg, 95.3–184 mg/kg, and 42.6–80.7 mg/kg, respectively. Before processing, the fruits were stored at −20 °C for 3 months in the dark. The preparation was measured using a previously described method with minor modifications [4]. After the fruits were thawed, they were chopped and heated in water (fruit/water: 1:4 *v*/*v*) at 90 °C for 10 min. Subsequently, the heated fruit juice was passed through a 400-mesh filter to obtain a slightly yellow EWSJ. The filtrate contained a small amount of the precipitate. The pH of the obtained juice was adjusted to approximately 7 via the dropwise addition of 0.94 M NaHCO_3_ aqueous solution. After preparation, the sugar, acid, and protein content of EWSJ were 82–120 mg/kg, 19.1–36.8 mg/kg, and 7.90–16.10 mg/kg, respectively. The juice was then stored at −20 °C in the dark until further analysis.

#### 2.1.2. Activation of *Bifidobacterium*

*B. animalis* subsp.* lactis* HN-3 (B.an3, CCTCC NO: M20221023) was isolated from human feces samples in the Hainan province of China by the technical innovation team of Characteristic Probiotic Resources and Dairy Technology Industrialization, Microbiology Lab, Shihezi University. B.an3 was activated and propagated statically in sterile MRS broth containing 0.05% (*w*/*v*) L-cysteine and 0.005% (*w*/*v*) mupirocin via stationary culture in Don Whitley Scientific DG250 Anaerobic Workstation (Don Whitley Scientific, West Yorkshire, UK) under strict anaerobic conditions in the dark for 48 h at 37 °C.

### 2.2. Fermentation and Determination of Viable Counts

This procedure was performed according to the method of Wang et al., with minor modifications [6]. Before ESWJ fermentation by B.an3, frozen ESWJ was sterilized in an autoclave (Shanghai Boxun Industrial Co., Ltd., Shanghai, China) at 90 °C for 10 min. For separate (1 × 10^8^ CFU/mL, 2% (*v*/*v*)) fermentations, B.an3 was inoculated with sterilized ESWJ and incubated at 37 °C for 48 h. The bacterial viable counts were determined at 0, 12, 24, 36, and 48 h during fermentation. The cell density was measured at 600 nm using a UV-vis spectrophotometer (PERSEE, Beijing, China). Furthermore, at each of the six-time points, 10 mL aliquots of the fermented juice and control juice (without *B. animalis* subsp.* lactis* HN-3) were immediately frozen in liquid nitrogen, transferred to a –80 °C deep freezer, and stored in the dark for metabolomic analysis.

### 2.3. Measurement of Soluble Solids and pH

Total soluble solids content (TSSC) in EWSJ was measured using a previously reported method with minor modifications [13]. The soluble solid content was determined using a digital handheld Brix meter (ATAGO, Tokyo, Japan). An aliquot (300 μL) of the fermented juice was dripped onto the Brix meter. The degree was the content of soluble solids. Control solutions were prepared using 300 µL of distilled water instead of fermented EWSJ. The pH was determined using a digital pH meter (Mettler-Toledo, Greifensee, Switzerland) [13].

### 2.4. Total Flavonoid Content (TFC)

TFC in the fermented EMS juice was determined using the aluminum chloride colorimetric method described by Sarker and Oba [14]. For this procedure, 500 µL of the fermented EWSJ was transferred to a test tube, and 1.5 mL of methanol, 0.1 mL of 10% aluminum chloride, 0.1 mL of 1 mol L^−1^ potassium acetate, and 2.8 mL of distilled water were added. After incubating for 30 min at room temperature, the absorbance of the reaction mixture was measured at 415 nm spectrophotometrically (HitachiU1800, Hitachi, Tokyo, Japan). Rutin was used as the standard compound, and TFC was expressed as mg of rutin equivalent (RE)/L of the solution.

### 2.5. Determination of the Antioxidant Capacity of EWSJ

#### 2.5.1. ABTS^+•^ Radical-Scavenging Ability

The antioxidant capacity of the fermented EWSJ was determined using the free-radical scavenger 2,2-binamine-di-3-ethylbenzothiazolin-6-sulfonic acid (ABTS^+•^) using a previously published method, with slight modifications [15]. The ABTS^+•^ radical cation was pre-generated by mixing a 7 mM solution of ABTS (0.8 mL, water) with a 2 mM solution of potassium persulfate (1.5 mL, water) in the dark for 16 h at 20 °C. Prior to the analysis, the resulting ABTS^+•^ solution was diluted with sterile water until an absorbance of 0.700 ± 0.005 (starting absorbance) at 734 nm was obtained. Subsequently, an aliquot (200 μL) of the fermented juice was added to a test tube and mixed with 800 μL of the diluted ABTS^+•^ solution, the absorbance of which was measured at 734 nm after mixing for exactly 6 min. Control solutions were prepared using 200 µL of distilled water instead of the fermented EWSJ. The ABTS^+•^ radical-scavenging antioxidant capacity, which was calculated using Equation (1) given below, was expressed as Trolox equivalents per 100 g of fresh fruit (μmol TE/100 g of fresh fruit).

(1) $$\mathrm{ABTS}\;\mathrm{radical}-\mathrm{scavenging}\;\mathrm{activity}\;(\%)=\left(1-\frac{A_{j}}{A_{0}}\right)\times 100\%$$

where A_0_ represents the absorbance of the control and A_j_ represents the absorbance of the ABTS^+•^ solution mixed with the fermented EWSJ.

#### 2.5.2. DPPH^•^ Radical-Scavenging Ability

The free radical-scavenging capacity of the EWSJ extracts was determined using the 2,2-diphenyl-1-picrylhydrazyl (DPPH) radical-scavenger assay according to the Brand-Williams method, with slight modifications [16]. Briefly, 2 mL of the fermented EWSJ was mixed with 0.5 mL of a 1 mM DPPH solution in 95% (*v*/*v*) ethanol and 0.5 mL of methanol, and the absorbance was measured using a UV-vis spectrophotometer at 517 nm after incubating for 30 min at room temperature in the dark. The percentage of scavenging activity, which was calculated using Equation (2) furnished below, was expressed as Trolox equivalents per 100 g of fresh fruit (μmol TE/100 g of fresh fruit).

(2) $$\mathrm{DPPH}\;\mathrm{radical}-\mathrm{scavenging}\;\mathrm{activity}\;(\%)=\left(1-\frac{A_{i}-A_{j}}{A_{c}}\right)\times 100\%$$

where A_c_ represents the absorbance of the DPPH solution mixed with methanol (control), A_i_ represents the absorbance of the fermented EWSJ mixed with the DPPH solution, and A_j_ represents the absorbance of the fermented EWSJ mixed with methanol.

### 2.6. Non-Targeted Metabolomic Analysis

#### 2.6.1. Metabolite Extraction

The samples (100 μL) were taken in Eppendorf tubes, resuspended in prechilled 80% methanol, and vortexed well. Then, the samples were incubated on ice for 5 min and centrifuged at 15,000× *g* for 20 min at 4 °C. The supernatant was diluted with LC-MS grade water to the final concentration containing 53% methanol. The samples were subsequently transferred to fresh Eppendorf tubes and centrifuged at 15,000× *g* for 20 min at 4 °C. Finally, the supernatant was injected into the LC-MS/MS system for analysis [17].

#### 2.6.2. UHPLC-MS/MS Analysis

UHPLC-MS/MS analyses were performed using a Vanquish UHPLC system (ThermoFisher, Osterode am Harz, Germany) coupled with an Orbitrap Q Exactive TMHF mass spectrometer (Thermo Fisher, Germany) in Novogene Co., Ltd. (Beijing, China) [18]. The samples were injected onto the HypesilGoldcolumn (100 × 2.1 mm, 1.9 μm) using a 17 min linear gradient at a flow rate of 0.2 mL/min. The eluents for the positive polarity mode were eluent A (0.1% FA in water) and eluent B (methanol). Those for the negative polarity mode were eluent A (5 mM ammonium acetate, pH 9.0) and eluent B (methanol). The solvent gradient was set as follows: 2% B, 1.5 min; 2–85% B, 3 min; 85–100% B, 10 min; 100–2% B, 10.1 min; and 2% B, 12 min. Q ExactiveTM HF mass spectrometer was operated in positive/negative polarity mode with a spray voltage of 3.5 kV, capillary temperature of 320 °C, sheath gas flow rate of 35 psi, auxiliary gas flow rate of 10 L/min, S-lens RF level of 60, and auxiliary gas heater temperature of 350 °C.

#### 2.6.3. Data Processing and Metabolite Identification

The raw data files generated using UHPLC-MS/MS were processed using Compound Discoverer 3.1 (CD3.1, ThermoFisher) to perform peak alignment, peak picking, and quantitation for each metabolite [19]. The main parameters were set as follows: retention time tolerance, 0.2 min; actual mass tolerance, 5 ppm; signal intensity tolerance, 30%; signal/noise ratio, 3; and minimum intensity, et al. Subsequently, peak intensities were normalized to the total spectral intensity. The normalized data were used to predict the molecular formula based on additive ions, molecular ion peaks, and fragment ions. The peaks were then matched with mzCloud (https://www.mzcloud.org/, which be open accessed on 15 March 2024), mzVault, and MassListdatabase to obtain accurate qualitative and relative quantitative results. Statistical analyses were performed using the statistical software R (R version R-3.4.3), Python (Python 2.7.6 version), and CentOS (CentOS release 6.6). When the data were not normally distributed, they were standardized according to the formula “sample raw quantitation value/(the sum of sample metabolite quantitation value/the sum of QC1 sample metabolite quantitation value)” to obtain relative peak areas. Compounds whose coefficients of variation of relative peak areas in quality control samples were >30% were removed, and finally, the identification and relative quantification results of the metabolites were obtained.

### 2.7. Statistical Analysis

The results of viable counts, pH, soluble solids, total flavonoids and antioxidant capacity of EWSJ were expressed as the mean (±standard deviation) of three replications. The non-targeted metabolomic analysis was six replications. Data were analyzed using IBM SPSS Statistics version 26 (SPSS Inc., Chicago, IL, USA). To compare the differences in physicochemical properties, a one-way analysis of variance was performed using the Tukey test. These metabolites were annotated using the KEGG database (https://www.genome.jp/kegg/pathway.html), HMDB database (https://hmdb.ca/metabolites), and LIPIDMaps database (http://www.lipidmaps.org/). These database all be open accessed on 15 March 2024.

## 3. Results

### 3.1. Viable Counts and Physicochemical Properties during B.an3 Fermentation

**Figure 1:**
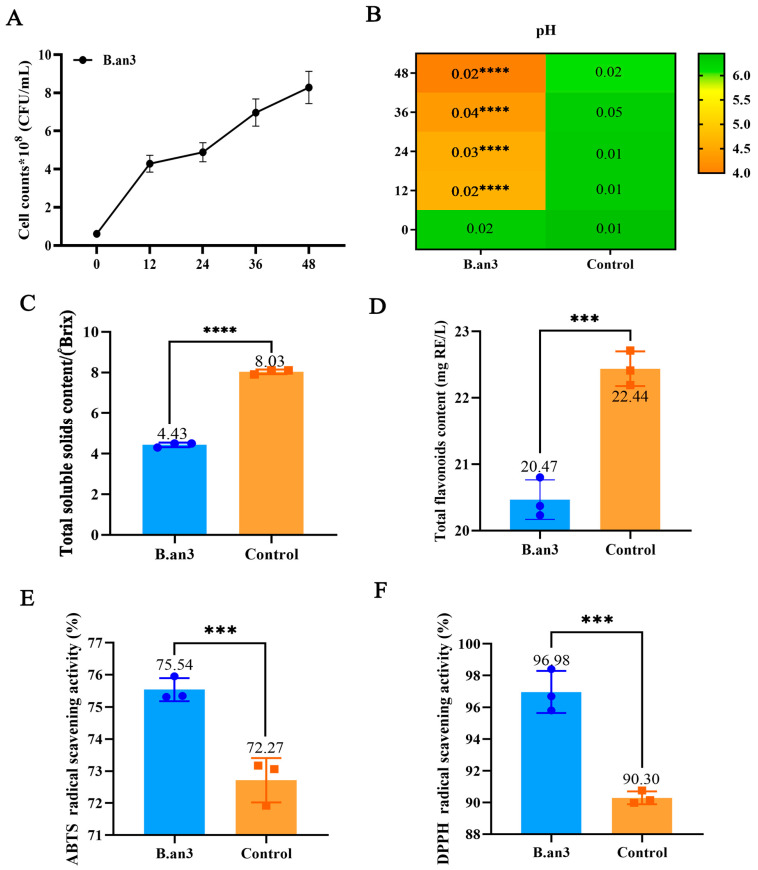
The dynamics of viable counts (**A**) and pH (**B**) in (Fermented) fermented *Elaeagnus moorcroftii* Wall.*ex Schlecht.* juice (EWSJ) by *B. animalis* subsp.* lactis* HN-3 (B.an3) and (Control) unfermented *Elaeagnus moorcroftii* Wall.*ex Schlecht.* juice (EWSJ) during 48 h. Data are expressed as SD. Total soluble solids content (TSSC) (**C**), total flavonoid content (TFC) (**D**), and antioxidant capacity assays ABTS (**E**) and DPPH (**F**), as well as free radical scavenging activity of (Fermented) fermented EWSJ by B.an3 and (Control) unfermented EWSJ at 48 h of fermentation at 37 °C. Values represent averages of triplicate measurements. Data are expressed as mean ± SD. Asterisks illustrate statistical significance. *** 0.0001 < *p* < 0.001, **** *p* < 0.0001.

The population of probiotics in fermented juices is a key indicator for the development of probiotic-fermented functional foods [1]. As depicted in Figure 1A, the cell count of *B. animalis* subsp.* lactis* HN-3 (B.an3) was the highest (8.22 × 10^8^ CFU/mL) in EWSJ after 48 h of fermentation. This population exceeded the standard for commercial probiotic-fermented food production (10^7^ CFU/mL) [4]. The growth of *Bifidobacterium* was positively correlated with the energy and carbohydrate consumption patterns. During the growth of this strain, energy is produced through the conversion of carbohydrates into organic acids while producing Adenosine triphosphate (ATP) [20,21]. Therefore, the B.an3-fermented EWSJ has the potential to be developed as a functional food.

The pH of the B.an3-fermented EWSJ decreased considerably after 12 h of fermentation and reached 4.00 at 48 h (Figure 1B). In this study, compared with unfermented EWSJ, a significant decrease in °Brix from 8.03 ± 0.12 to 4.43 ± 0.11 was observed during B.an3 fermentation (as presented in Table 1 and Figure 1C). Meanwhile, the °Brix of the fermented juice should strike a balance between sugar consumption and organic acid production [22]. Combined with the previously described alterations in pH, a large amount of carbohydrates was converted to produce a high level of acid in EWSJ during B.an3 fermentation.

As listed in Table 1, the TFC of EWSJ fermented with B.an3 was lower than that of unfermented EWSJ. After 48 h of fermentation, the TFC was significantly reduced from 22.44 mg GAE/L to 20.47 mg GAE/L (*p* < 0.01) (Figure 1D). Nonetheless, B.an3-fermented EWSJ exhibited strong ABTS and DPPH radical scavenging activity. Compared with unfermented EWSJ, the ABTS radical scavenging activity was increased by 3.27% (Figure 1E), and the DPPH radical scavenging activity was significantly increased by 6.68% (*p* < 0.01) (Figure 1F). A similar trend has been reported in B. lactis BB-12-fermented red pitaya pulp juice. Although the TFC of the fermented juice was lower, the fermented juice had higher antioxidant activity than the unfermented juice [23]. Antioxidants reduce the effects of free radicals on tumors, inflammation, cell growth, and DNA damage by scavenging reactive free radicals. Flavonoids, carbohydrates, phenols, amino acids, fatty acids, and terpenes can scavenge free radicals [24]. Therefore, it is important to further explore the relationship between significant changes in growth, pH, total soluble solids content, antioxidant activity, and the biotransformation of metabolites by B.an3.

### 3.2. Metabolite Analysis of EWSJ Fermented by B. animalis subsp. lactis HN-3

#### 3.2.1. Metabolic Responses to the Fermentation of EWSJ

**Figure 2:**
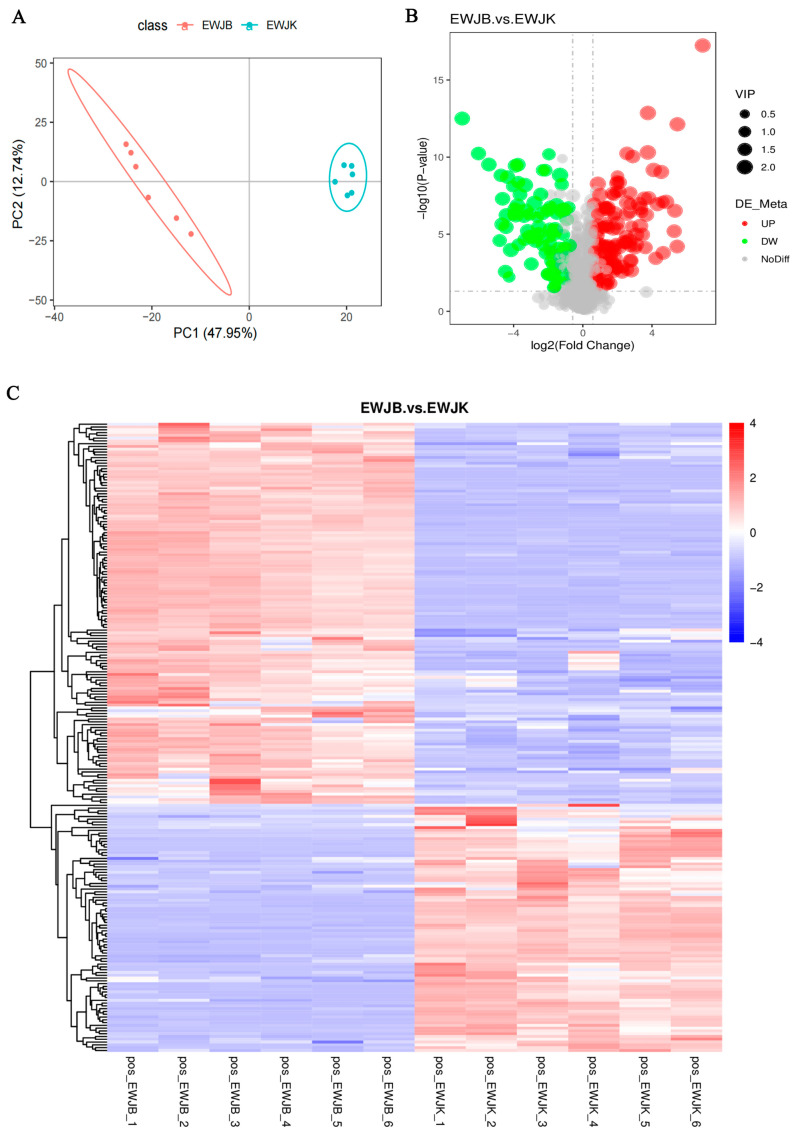
(**A**) Principal component analysis (PCA) of metabolic profiles in all samples (six biological replications). (**B**) V-plot of (Fermented) fermented *Elaeagnus moorcroftii* Wall.*ex Schlecht.* juice (EWSJ) by *B. animalis* subsp.* lactis* HN-3 (B.an3) and (Control) unfermented *Elaeagnus moorcroftii* Wall.*ex Schlecht.* Juice (EWSJ). The red color represents upregulation, and the green color represents downregulation; (**C**) The classification heat map of total differential metabolites of two EWSJ samples.

The metabolite profiles of B.an3-fermented EWSJ and unfermented EWSJ were analyzed using UHPLC-MS, and a total of one thousand three hundred and seventy-four metabolites were identified (Table S1), which included the following: two hundred and fifty-four lipids and lipid-like molecules; one hundred and sixty-nine organic acids and derivatives; one hundred and fifty phenylpropanoids and polyketides; one hundred and forty-four organoheterocyclic compounds; one hundred and twenty-two organic oxygen compounds; ninety-three benzenoids; fifty-eight nucleosides, nucleotides, and analogs; fourteen organic nitrogen compounds; thirteen alkaloids and derivatives; one homogeneous nonmetal compound; one hydrocarbon derivative; one lignan, neolignan, and related compounds; and three hundred and eighty others. Among these metabolites, the first five classes of substances with quantities > 100 include metabolites produced during the growth of the strain and functionally active substances that initiate changes in the antioxidant activity of the EWSJ. For example, lipids and lipid-like molecules contain fatty acids; organic acids and derivatives contain amino acids; phenylpropanoids and polyketides contain flavonoids; organic oxygen compounds contain carbohydrates. As shown in the principal component analysis (Figure 2A), the individual points in the plot of both the fermented and unfermented EWSJ samples were divided into two regions, and the separation between the fermented and control EWSJ samples was significant, which indicated that the metabolite content in EWSJ changed gradually after B.an3 fermentation. The values of R2Y (1.00) and Q2Y (0.98) in the partial least squares-discriminant analysis (PLS-DA) were close to 1, indicating that the metabolic model was stable and reliable. Meanwhile, the vector values of R^2^ (0.0, 0.89) and Q^2^ (0.0, −0.0.84) from these two EWSJ samples indicated that the PLS-DA model was not overfitting (as shown in Figures S1 and S2). Consequently, the present experimental model allows for further differential analyses.

#### 3.2.2. Overview of Differential Metabolites after the Fermentation of EWSJ

Differential metabolites were screened based on the criteria of VIP > 1.0, FC > 1.5, or FC < 0.667 and *p*-value < 0.05. Figure 2B presents the volcano plots of differential metabolites detected using various ionic modes. A total of 370 metabolites were found to be statistically significant in fermented and nonfermented EWSJ, of which 187 were upregulated (red dots) and 183 were downregulated (green dots). Hierarchical cluster analysis was used to classify metabolites with similar characteristics and identify intergroup variations in metabolites (Figure 2C). The color sequence from blue to red indicates the order of metabolite reduction.

**Figure 3:**
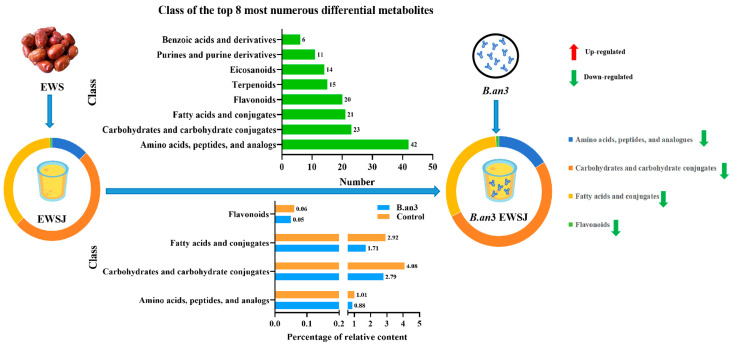
The classes of the top 8 numerous differential metabolites of (Fermented) fermented *Elaeagnus moorcroftii* Wall.*ex Schlecht.* juice (EWSJ) by *B. animalis* subsp.* lactis* HN-3 (B.an3) and (Control) unfermented *Elaeagnus moorcroftii* Wall.*ex Schlecht.* iuice (EWSJ) and the percentage of relative content of flavonoids, fatty acids, carbohydrates, and amino acids in two EWSJ samples. The red color (↑): represents increased. The green color (↓): represents a decrease.

Compared with the unfermented juice, amino acids, peptides, and analogs; carbohydrates and their conjugates; fatty acids and their conjugates; flavonoids; eicosanoids; purines and purine derivatives; terpenoids, benzoic acids, and derivatives were the classes of the top eight numerous differential metabolites of fermented EWSJ by B.an3. As shown in Table 2 and Figure 3, the four groups of substances that were most significantly decreased by bifidogenic fermentation included amino acids, peptides, and analogs; carbohydrates and their conjugates; fatty acids and their conjugates; and flavonoids. This result corresponds to the decrease noted in TSSC and flavonoid content with the use of physicochemical indicators. According to previous reports, these four groups of metabolites act as the main antioxidant active components in EWS, and the decrease in their content should, therefore, reduce the antioxidant activity of the juice. However, the antioxidant activity of B.an3 fermented juice increased in the present study. Therefore, 107 specific differential metabolites were examined further.

**Figure 4:**
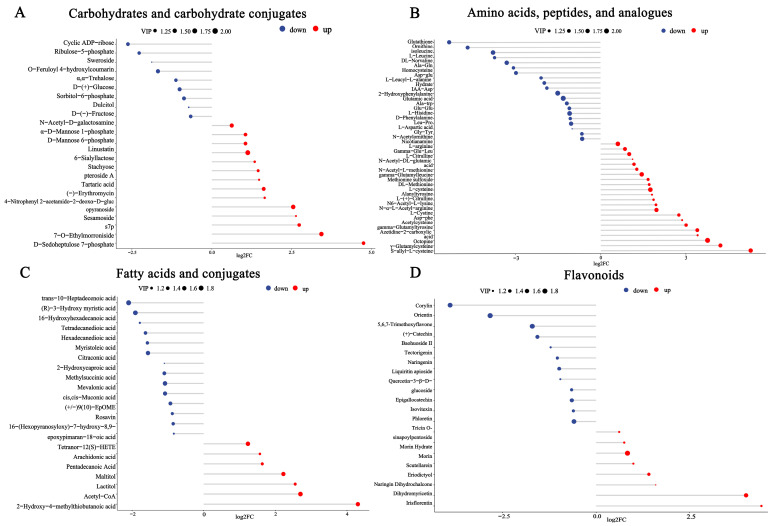
The regulation of significantly different flavonoids, fatty acids, carbohydrates, and amino acids in (Fermented) fermented *Elaeagnus moorcroftii* Wall.*ex Schlecht.* juice (EWSJ) by *B. animalis* subsp.* lactis* HN-3 (B.an3) and (Control) unfermented *Elaeagnus moorcroftii* Wall.*ex Schlecht.* Juice (EWSJ). The regulation (**A**) of carbohydrates. The regulation (**B**) of amino acids. The regulation (**C**) of fatty acids. The regulation (**D**) of flavonoids. The red color represents upregulation, and the blue color represents downregulation.

The regulation of carbohydrates, amino acids, fatty acids, and flavonoids were shown in Figure 4A–D, respectively. With regard to carbohydrates, there were twenty-three differential metabolites: fourteen metabolites were upregulated, and nine metabolites were downregulated. The upregulated carbohydrates have more reactive oxygen on benzene rings, such as D-mannose 6-phosphate, D-sedoheptulose 7-phosphate, and glucose-1-phosphate. A total of 42 differential metabolites were identified in the class of amino acids, peptides, and analogs; 22 metabolites were upregulated, and 20 metabolites were downregulated. The upregulated amino acids contain sulfhydryl or hydroxy groups, such as acetylcysteine, L-arginine, L-citrulline, L-aspartic acid, L-cysteine, L-cystine, S-allyl-L-cysteine, octopine, DL-methionine, and nicotianamine. With regard to fatty acids, there were twenty-one differential metabolites: seven metabolites were upregulated, and four were downregulated. Of the upregulated fatty acids, arachidonic acid, 2-Hydroxy-4-methylthiobutanoic acid, and Maltitol were unsaturated fatty acids, short-chain fatty acids, and fatty acyl glycosides, respectively. Then, twenty differential metabolites were identified in the class of flavonoids—eight metabolites were upregulated, and twelve metabolites were downregulated—the flavonoids were in the form of hydrolyzed glycosides and methoxy conjugates.

Thus, in this study, we speculated that the increase in the antioxidant activity is related to the structure of the upregulated substances and that the structural changes result from the transformation and metabolism of these substances. Accordingly, a further search for metabolic transformation relationships of substances was undertaken.

### 3.3. Major Metabolism during the Fermentation of EWSJ

**Figure 5:**
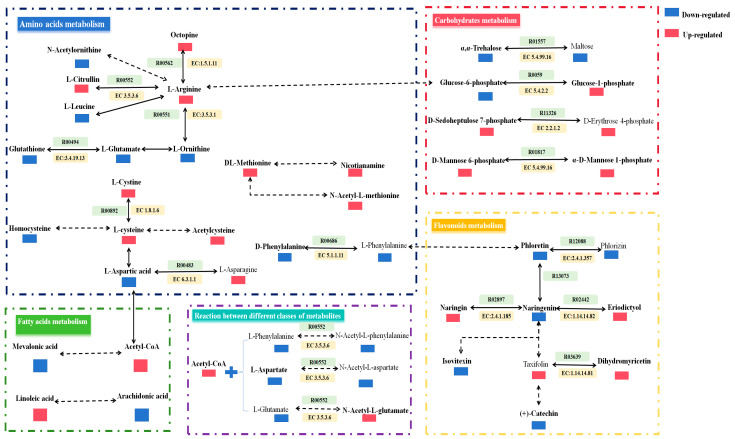
The changes in metabolites mapped to the metabolic pathways in two EWSJ samples. The red blocks (↑) represent an increase. The green blocks (↓) represent a decrease. The full line represents direct conversion, and the dotted line represents indirect conversion. The pathways were retrieved from the KEGG database and modified, including carbohydrate metabolism, fatty acid metabolism, amino acid metabolism, flavonoid metabolism, conversion reactions between different classes of metabolites, and reaction and transformation enzymes.

Using the KEGG database, the differential metabolites of the four classes were annotated to further analyze the metabolism and mechanisms of significant changes in these substances after B.an3 fermentation. These biotransformation reactions and their regulation were summarized, and the enzymes involved in the transformation of the metabolites were predicted, as presented in Figure 5. Generally, there are four types of metabolism, including amino acid, carbohydrate, fatty acid, and flavonoid metabolism. As shown in Figure 5, amino acid metabolism had the highest number of direct or indirect transformations between differential metabolites, followed by flavonoid metabolism, carbohydrates metabolism, and finally, fatty acid metabolism. In addition, reactions between different types of substances were involved.

After fermentation, L-arginine and L-cysteine were the central metabolites of amino acid metabolism. L-arginine has a direct or indirect translational relationship with L-citrulline, L-leucine, N-acetylornithine, octopine, and L-ornithine. Similar to L-arginine, L-cysteine also undergoes direct and indirect transformations with L-cystine, homocysteine, acetylcysteine, and L-aspartic acid. Moreover, a direct transformation was noted between the decrease in the content of D-phenylalanine and a slight decrease in the content of L-phenylalanine. In addition, an indirect transformation relationship was also noted between the increase in the content of DL-methionine and the increase in the content of nicotianamine and N-acetyl-L-methionine. According to the metabolite regulation analysis and KEGG metabolic pathway analysis, there are seven classes of direct conversion reactions, including three hydrolysis reactions, two reduction reactions, one isomerization reaction, and one other class of reactions. The enzymes within this metabolism that were implicated in the biotransformation reactions include hydrolases, oxidoreductases, isomerases, and ligases.

The second most dominant metabolism was flavonoid metabolism. Naringenin, as the key metabolite in the flavonoid metabolic pathway, had a direct or indirect translational relationship with phloretin, naringin, eriodictyol, iso-vitexin, and taxifolin. Furthermore, the increase in the content of taxifolin leads to an indirect transformation with the decrease in the content of (+) catechin and leads to a direct transformation with the increase in the content of dihydromyricetin. Meanwhile, based on an analysis of the metabolic pathway data by the KEGG database, these four reactions might also be catalyzed by glycosyltransferases and reductases. Interestingly, the corresponding enzymes involved in the reaction between naringenin and phloretin have not been marked in the KEGG database. Therefore, it would be advantageous to further study the enzymes implicated in these transformations.

The third most dominant metabolism was carbohydrate metabolism. The contents of many carbohydrates in the EWSJ increased after fermentation. In the four direct conversion reactions of carbohydrates, three isomerization reactions and one other reaction were included. Meanwhile, based on an analysis of the metabolic pathway data by the KEGG database, these four reactions might also be catalyzed by isomerases and transferases.

When compared to amino acids, flavonoids, and carbohydrates metabolism, the metabolite transformations in the fatty acid metabolism were all indirect syntheses. Thus, the transformation of fatty acids was explored in further experiments. Acetyl-CoA was identified as an important metabolite as it not only converted into other fatty acids but also reacted with metabolites of other classes, such as L-phenylalanine, L-aspartate, and L-glutamate to form the corresponding N-acetyl-L-phenylalanine, N-acetyl-L-aspartate, and N-acetyl-L-glutamate, respectively. These three reactions were all catalyzed by transferases.

Interestingly, six metabolites served as bridges between the different metabolic pathways, namely, L-arginine, L-aspartic acid, L-phenylalanine, acetyl-CoA, glucose-6-phosphate, and phloretin. According to the biosynthesis pathways of plant secondary metabolites, glucose-6-phosphate can be interconverted with L-arginine via the pentose phosphate pathway, and L-arginine can be interconverted with acetyl-CoA. Cho et al. observed that L-arginine stimulated the production of glucose-6-phosphate by mediating the ubiquitination of glucokinase [25]. Similarly, L-aspartic acid stimulated acetylation by reacting acetyl-CoA with amino acids to form acetylated amino acids. Furthermore, L-phenylalanine formed coumarins in the biosynthesis of phenylpropanoids. In the study by Son et al., trans-cinnamic acid was produced by whole-cell bioconversion from L-phenylalanine [26]. Moreover, the coumarins were converted to chalcone (such as phloretin) via the flavonoid metabolism pathway.

B.an3 fermentation included the conversion of carbohydrates, amino acids, fatty acids, and flavonoids. After the transformations of their metabolic precursors, the contents of metabolites with the high antioxidant activity structure were significantly increased when compared to that in the unfermented EWSJ. Their increased production during fermentation helped manifest an improved overall antioxidant capacity of the fermented EWSJ.

## 4. Discussion

The fruit of EWS was determined to be an excellent substrate for fermentation as its naturally occurring metabolites were not only converted by the probiotics into biologically active metabolites but also provided nutrients for probiotic growth. Nevertheless, the specific metabolic mechanisms of B.an3 on the nutrients and functionally active substances in EWSJ have not been completely elucidated. Therefore, the metabolic and translational mechanisms of B.an3 fermentation were investigated to enhance the antioxidant activity of EWSJ via non-targeted metabolomics. In conclusion, B.an3 fermentation can significantly alter the metabolite content of EWSJ, especially carbohydrates, amino acids, fatty acids, and flavonoids. Meanwhile, the functional activity of B.an3 fermented EWSJ was significantly changed.

### 4.1. Effects on the Functional Activity during the Fermentation of EWSJ

#### 4.1.1. Impact of B.an3 Growth

*Bifidobacterium animalis* subsp.* lactis* HN-3 showed good growth in fermented EWSJ. Some of the significantly different carbohydrates and fatty acids promoted the growth of strains. It is worth noting that of the various upregulated amino acids, acetyl-CoA is not only a key metabolite with antioxidant activity but also participates in several metabolic pathways that regulate cell growth, senescence, and death [27]. Among these metabolites, D-sedoheptulose 7-phosphate, a signal transduction molecule, is important for the infection, colonization, and immune recognition of gram-negative bacteria and is also a component of various natural products with fascinating bioactivities from gram-positive bacteria [20]. The other upregulated metabolites also demonstrate functional activities. For instance, α-D-mannose 1-phosphate enhances the supply of ATP-stored energy to promote bacterial growth [21]; hence, the good growth of B.an3 was associated with these metabolites.

#### 4.1.2. Antioxidant

*Bifidobacterium animalis* subsp.* lactis* HN-3 significantly improved the overall antioxidant capacity of the EWSJ. As previously mentioned, the ABTS-free radical-scavenging rate correlated with free radical-scavenging capacity. In contrast, the DPPH radical-scavenging rate correlated with metal-chelating ability. Accordingly, we have discussed the four classes of substances based on these two ways of scavenging free radicals.

##### Free Radical Scavenging Capacity

Carbohydrates, amino acids, flavonoids, and fatty acids were mainly obtained through scavenging free radical capacity to improve the antioxidant activity.

In this study, the upregulated carbohydrates displayed strong antioxidant activity in B.an3-fermented EWSJ and included D-mannose 6-phosphate, D-sedoheptulose 7-phosphate, and glucose-1-phosphate. Among these metabolites, their structures have more reactive oxygen molecules on benzene rings to react with free radicals. However, among the downregulated metabolites, the downregulation of α,α-trehalose reduced the sweetness of the juice and can hence be used as an antioxidant agent [28].

As natural antioxidants, amino acids contain sulfhydryl or hydroxy groups that inactivate free radicals owing to resonance delocalization throughout the phenolic ring structure [8]. Of the upregulated amino acids, acetylcysteine, L-arginine, L-citrulline, L-aspartic acid, L-cysteine, L-cystine, S-allyl-L-cysteine, octopine, DL-methionine, and nicotianamine have been reported to be used as antioxidants in clinical treatment as well as agricultural production. What is more, the upregulated substances included several dipeptides formed from different amino acids, such as N-acetyl-L-glutamate and N-acetyl-L-methionine. These metabolites possessed a π conjugate or a thiol group in their molecular structure; the π conjugate stabilizes free radicals by their delocalization, and two thiol groups form disulfide bonds in the antioxidant process [9].

Flavonoids are secondary metabolites with diverse chemical structures and functions and are widespread among fruits [29]. Flavones exhibit a high antioxidant activity because they contain the structure of A-ring and C-ring with a 4-oxo group and a double bond between C-2 and C-3 [29]. Etta et al. reported the ability of eri-odictyol to enhance antioxidant activity [4]. However, the downregulated flavonoids mostly included glycosides, methylated flavonoids, and methyl ester conjugates [29], such as liquiritin apioside, quercetin-3β-D-glucoside, and phloretin.

Unsaturated fatty acids, short-chain fatty acids, and fatty acyl glycosides exhibit strong antioxidant activity [7]. Of the upregulated fatty acids, arachidonic acid, 2-Hydroxy-4-methylthiobutanoic acid, and Maltitol were unsaturated fatty acids, short-chain fatty acids, and fatty acyl glycosides, respectively.

##### Metal-Chelating Ability

Some amino acids and flavonoids possess the metal-chelating ability. For example, the downregulated L-glutamic acid and L-ornithine demonstrated antioxidant activity by chelating prooxidative metals [30]. The contents of L-glutamic acid and L-ornithine decreased after B.an3 fermentation, which directly contributed to the elevated antioxidant activity of fermented EWSJ.

Of the various flavonoids, flavonols with multiple hydroxyl groups display the strongest antioxidant activity [29]. The upregulated dihydromyricetin exhibited this structural feature and has 4-oxo groups and -OH groups near C-3 and C-5. This structure can promote hydroxyl groups to generate unstable electrons to scavenge free radicals by affecting the ionic domains around flavonols [6].

*Bifidobacterium animalis* subsp.* lactis* HN-3 possibly improved the antioxidant capacity of the EWSJ through three different mechanisms: (1) metabolites with antioxidant active structures were increased in the EWSJ during fermentation by *Bifidobacterium animalis* subsp.* lactis* HN-3. (2) Downregulated metabolites: a few of them are strong antioxidants and were consumed during fermentation to enhance the antioxidant activity. (3) The other downregulated metabolites with low antioxidant activities were transformed into the corresponding metabolites, most of which entailed strong antioxidant properties. Further validation of the specific antioxidant mechanism was needed. We speculated the abovementioned three mechanisms and further analyzed the specific mechanisms for enhancing antioxidant activity.

### 4.2. Major Transformation during the Fermentation of EWSJ

B.an3 fermentation converted carbohydrates, amino acids, fatty acids, and flavonoids via 18 direct conversion reactions. These included four reduction reactions, three hydrolysis reactions, four isomerization reactions, two deglycosylation reactions, and five other reactions. The reaction types, precursors, products, and predicted en-zymes are summarized in Table 3 and are discussed in the forthcoming sections.

#### 4.2.1. Reduction

Reduction reactions are the action of oxidoreductases, which mainly increase the number of hydroxy groups and decrease the number of methoxy groups. For amino acids, the predicted enzymes acting on L-cystine and octopine in the two reduction reactions were oxidoreductases to increase hydroxy, namely cystine reductase (EC 1.8.1.6) and D-octopine dehydrogenase (EC 1.5.1.11). In *Bifidobacterium*, *Bifidobacterium longum* subsp. *Infantis* produced amino acid oxidoreductase [31]. For flavonoids, naringenin and taxifolinfen were converted by flavonoid 3′-monooxygenase (EC 1.14.14.82) and flavonoid 3′,5′-hydroxylase (EC 1.14.14.81), respectively. These two enzymes increase the hydroxy of taxifolinfen and decrease the methoxy of naringenin. Langa et al. identified reductases in *Bifidobacterium pseudocatenulatum* INIA P815 that converted equol to 5-hydroxy-equol [32].

#### 4.2.2. Hydrolysis

Hydrolase is the main conversion enzyme of hydrolysis. Similar to reductase, hydrolase also increases the number of hydroxy groups. For example, the predicted hydrolysis enzymes for the transformation of the precursor substances L-arginine, L-ornithine, and glutathione were arginine deiminase (EC 3.5.3.6), arginase (EC 3.5.3.1), and glutathione hydrolase (EC 3.4. 19.13), respectively. In a previous study, Thakker et al. reviewed the isolation of arginine deiminase from *Bifidobacterium bifidum* CGMCC 15,068 and *Bifidobacterium longum* 420 for the production of anticancer drugs [33].

#### 4.2.3. Isomerization

Isomerization changes the position of the existing group by isomerases. Therefore, isomerases transform the oxygen and sulfhydryl groups on the reactive sites so as to react with free radicals. The conversion of L-phenylalanine to D-phenylalanine occurred in the presence of the isomerase phenylalanine racemase (EC 5.1.1.11). Isomerases that metabolize amino acids to have more sulfhydryl were detected in *Bifidobacterium bifidum*, and *Bifidobacterium* breve produced isomerases capable of converting free lin-oleic acid to conjugated linoleic acid [34,35]. Meanwhile, by the action of isomerases (phosphomannomutase (EC 5.4.2.8), maltose al-pha-D-glucosyltransferase (EC 5.4.99.16), and phosphoglucomutase (EC 5.4.99.16)), D-mannose 6-phosphate, α,α-trehalose, and glucose-6-phosphate were converted to the corresponding α-D-mannose 1-phosphate, maltose, and glucose-1-phosphate, respectively. *Bifidobacterium adolescentis* produced the isomerase to metabolize carbohydrates to have more reactive oxygen on benzene rings [36].

#### 4.2.4. Deglycosidation

Deglycosidation was mainly detected in flavonoids. Phlorizin and naringenin, as flavonoid glycosides, were transformed to their aglycone via the action of phloretin 2′-O-D-glucosyltransferase (EC 2.4.1.357) and naringenin 7-O-glucosyltransferase (EC 2.4.1.185). Deglycosidation increases the number of hydroxyl groups by removing glycosides to generate unstable electrons, which then promotes hydrogen donation and electron transfer so as to scavenge free radicals. Meanwhile, *Bifidobacterium* possesses a rich glycosyltransferase system [37]. Moreover, in several past studies, *Bifidobacterium* has been reported to produce α-glucosidase, β-glucosidase, and α-rhamnosidase to remove glycosides [38].

#### 4.2.5. Others

Among the other reactions, the predicted enzymes mainly included ligases and transferase enzymes. The conversion of L-aspartic acid to L-asparagine was a function of ligases (L-asparagine synthetase (EC 6.3.1.1)). Fausta et al. observed the gene expression of ligases in *Bifidobacterium bifidum* PRL2010 [39]. However, D-sedoheptulose 7-phosphate was converted to D-erythrose 4-phosphate by the action of dihydroxyacetone transferase (EC 2.2.1.2). Lin et al. detected 10 carbohydrate-active enzyme gene families (GT51, GH13_32, GH26, GH42, GH121, GH3, AA3, CBM46, CE2, and CE6) that promoted the metabolism of carbohydrates in *Bifidobacterium pseudocatenulatum* isolated from humans [40]. Furthermore, there were transformation reactions between different classes of metabolites. All three reactions were other reactions, and all of them involved the binding of amino acids to acyl groups by the action of transferase. The transferases involved in these three reactions were phenylalanine N-acetyltransferase (EC 2.3.1.53), aspartate N-acetyltransferase (EC 2.3.1.17), and amino-acid N-acetyltransferase (EC 2.3.1.1). Acetyltransferase was isolated from *Bifidobacterium longum*, and this enzyme increased the amount of ornithine produced in fermented sunsik [41].

Overall, it was hypothesized that the increase in the antioxidant activity of B.an3-fermented EWSJ was due to specific enzymes produced by the strain acting on carbohydrates, amino acids, fatty acids, and flavonoids. Subsequent to the action of *Bifidobacterium* enzymes, the downregulated precursors with low antioxidant activity were converted via reduction, hydrolysis, isomerization, deglycosidation, and other reactions to generate the corresponding upregulated products with high antioxidant activity. *Bifidobacterium* produces transforming enzymes that act on specific sites that can be modified to produce products with better functional activities or applied for the batch production of potential drugs or functional fermented beverages. Nevertheless, the detailed mechanisms of specific biotransformations and transforming enzymes remain to be unraveled and investigated further. Future studies should, therefore, include multi-omics and validation of bacterial enzyme production to facilitate the comprehensive examination of the associations between enzymes inherent in the *Bifidobacterium* species and their metabolites.

## 5. Conclusions

This study identified the changes in carbohydrates, amino acids, fatty acids, and flavonoids upon the fermentation of EWSJ by *B. animalis* subsp.* lactis* HN-3 using non-targeted metabolomic analysis and investigated that antioxidant activity was significantly enhanced in B.an3-fermented EWSJ, which was mostly related to considerable alterations in the structure of carbohydrates, amino acids, fatty acids, and flavonoids of *Bifidobacterium* enzymes through reduction, hydrolysis, isomerization, deglycosidation, and others. However, the specific mechanisms of the metabolic transformations remain unclear without validation and a broader range of research in one strain of *Bifidobacterium* and one type of juice. Thus, the association between the enzymes inherent in the *Bifidobacterium* species and the metabolites needs to be more intensely investigated. This research provides a strong theoretical basis for the development of novel *Bifidobacterium*-fermented plant-derived products with enhanced functional activity. What is more, *Bifidobacterium* produces transforming enzymes that act on specific sites, which can be modified to produce products with better functional activity or applied to the batch production of potential drugs or functional fermented beverages.

## Figures and Tables

**Table 1:** The dynamics of physicochemical properties of *Elaeagnus moorcroftii* Wall.*ex Schlecht.* juice (EWSJ) fermented by *Bifidobacterium animalis* subsp.* lactis* HN-3 (B.an3).

Property	B.an3 Fermented EWSJ	Unfermented EWSJ
TSSC/(°Brix)	4.43 ± 0.11 ****	8.03 ± 0.12
TFC/(mg RE/L)	20.47 ± 0.03 ***	22.44 ± 0.02
ABTS/(%)	75.54 ± 0.01 ***	72.27 ± 0.01
DPPH/(%)	96.98 ± 0.01 ***	90.30 ± 0.01

Note: The soluble solids content (TSSC); Total flavonoid content (TFC); ABTS radical scavenging activity (ABTS); DPPH radical scavenging activity (DPPH). The value was mean ± std. *** 0.0001 < *p* < 0.001, **** *p* < 0.0001.

**Table 2:** The peak area of classes on the top 8 numerous differential metabolites of (Fermented) fermented *Elaeagnus moorcroftii* Wall.*ex Schlecht.* juice (EWSJ) by *B. animalis* subsp.* lactis* HN-3 (B.an3) and (Control) unfermented *Elaeagnus moorcroftii* Wall.*ex Schlecht.* juice (EWSJ).

Class	B.an3 Fermented EWSJ	Unfermented EWSJ
Amino acids, peptides, and analogs	10,872,660,962 ± 795,836,210.20 ***	13,443,673,365 ± 1,362,572,160.00
Carbohydrates and carbohydrate conjugates	34,615,427,601 ± 4,615,419,307.00 ****	54,506,204,164 ± 5,788,529,624.00
Fatty acids and conjugates	21,203,897,293 ± 558,396,702.60 ****	39,033,293,181 ± 3,012,718,214.00
Flavonoids	664,407,561 ± 19,242,552.98 ****	804,051,857 ± 48,092,759.05
Total peak area	1.24228 × 10^12^ ± 220,275,493.14	1.3371 × 10^12^ ± 112,031,813.99

Note: The value was the mean of six replications. Data are expressed as mean ± SD. Asterisks illustrate statistical significance. *** 0.0001 < *p* < 0.001, **** *p* < 0.0001.

**Table 3:** The main reaction types: reactive substances and predictive enzymes of fermented and unfermented *Elaeagnus moorcroftii* Wall.*ex Schlecht.* juice (EWSJ).

Type	Precursors	Products	Predictive Enzymes
Reduction (4)	L-Cystine (↑)	L-cysteine (↑)	Cystine reductase (EC 1.8.1.6)
	Octopine (↑)	L-Arginine (↑)	D-octopine dehydrogenase (EC 1.5.1.11)
	Naringenin (↓)	Eriodictyol (↑)	Flavonoid 3’-monooxygenase (EC 1.14.14.82)
	Taxifolin (↑)	Dihydromyricetin (↑)	Flavanoid 3’,5’-hydroxylase (EC 1.14.14.81)
Hydrolysis (3)	L-Arginine (↑)	L-Citrulline (↑)	Arginine deiminase (EC 3.5.3.6)
	L-Ornithine (↓)	L-Arginine (↑)	Arginase (EC 3.5.3.1)
	Glutathione (↓)	L-Glutamic acid (↓)	Glutathione hydrolase (EC 3.4.19.13)
Isomerization (4)	Glucose-6-phosphate (↓)	Glucose-1-phosphate (↑)	Phosphoglucomutase (EC 5.4.2.2)
	L-Phenylalanine (↓)	D-Phenylalanine (↓)	Phenylalanine racemase (EC 5.1.1.11)
	α, α-Trehalose (↓)	Maltose (↓)	Maltose alpha-D-glucosyltransferase (EC 5.4.99.16)
	D-Mannose 6-phosphate (↑)	α-D-Mannose 1-phosphate (↑)	Phosphomannomutase (EC 5.4.2.8)
Deglycosidation (2)	Phlorizin (↓)	Phloretin (↓)	Phloretin 2’-O-D-glucosyltransferase (EC 2.4.1.357)
	Naringenin (↓)	Naringin (↑)	Naringenin 7-O-glucosyltransferase (EC 2.4.1.185)
Others (5)	D-Sedoheptulose 7-phosphate (↑)	D-Erythrose 4-phosphate (↑)	Dihydroxyacetonetransferase (EC 2.2.1.2)
	L-Aspartic acid (↓)	L-Asparagine (↑)	L-asparagine synthetase (EC 6.3.1.1)
	L-Phenylalanine	N-Acetyl-L-phenylalanine	Phenylalanine N-acetyltransferase (EC 2.3.1.53)
	L-Aspartate	N-Acetyl-L-aspartate	Aspartate N-acetyltransferase (EC 2.3.1.17)
	L-Glutamate	N-Acetyl-L-glutamate	Amino-acid N-acetyltransferase (EC 2.3.1.1)

Note: (↑) represents upregulation after fermentation, and (↓) represents downregulation after fermentation.

## Data Availability

The original contributions presented in the study are included in the article/Supplementary Materials, further inquiries can be directed to the corresponding authors.

## Supplementary Materials

The following supporting information can be downloaded at https://www.mdpi.com/article/10.3390/foods13060926/s1. Figure S1: The score scatter plot of the OPLS-DA model; Figure S2: The values of R^2^Y and Q^2^ confirmed that the PCA and OPLS-DA models produced high-quality results; Figure S3: The Kegg Enrichment of significantly different flavonoids, fatty acids, carbohydrates and amino acids in (Fermented) fermented *Elaeagnus moorcroftii* Wall.*ex Schlecht.* juice (EWSJ) by *B. animalis* subsp.* lactis* HN-3 (B.an3) and (Control) unfermented *Elaeagnus moorcroftii* Wall.*ex Schlecht.* juice (EWSJ). The Kegg Enrichment (A) of carbohydrates; The Kegg Enrichment (B) of amino acids; The Kegg Enrichment (C) of fatty acids; The Kegg Enrichment (D) of flavonoids; Table S1: The 1374 metabolites of fermented and unfermented *Elaeagnus moorcroftii* Wall*.ex Schlecht.* juice were identified by non-targeted metabolomics.
