# Supplementary figures and images for: The Biotransformation and Influence on the Functional Activities of Metabolites during the Fermentation of Elaeagnus moorcroftii Wall.ex Schlecht. Juice by Bifidobacterium animalis subsp. lactis HN-3
Source: Foods. 2024 Mar 19;13(6):926. doi: 10.3390/foods13060926 (PMC10970000; doi:10.3390/foods13060926)

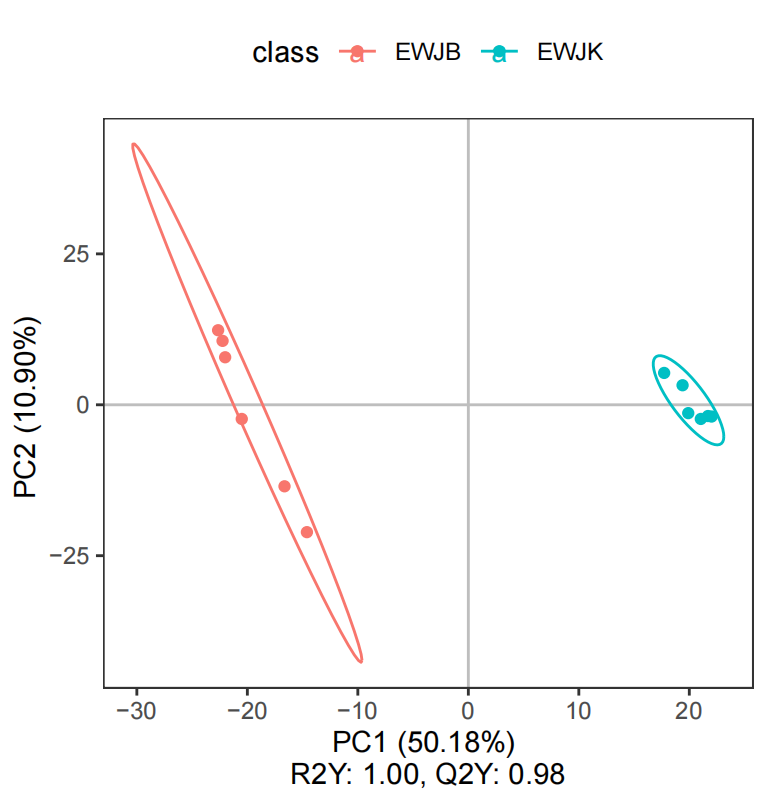

Supplement: Supplementary file 1 [file foods-13-00926-s001.zip › supplementary files/Fig. S1.tif]

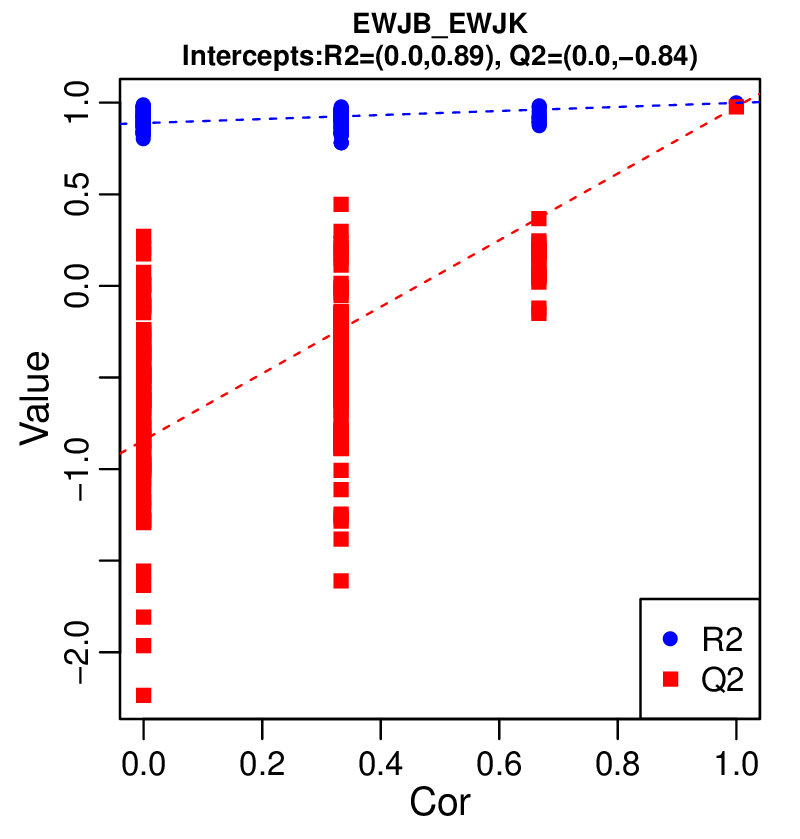

Supplement: Supplementary file 1 [file foods-13-00926-s001.zip › supplementary files/Fig. S2.png]

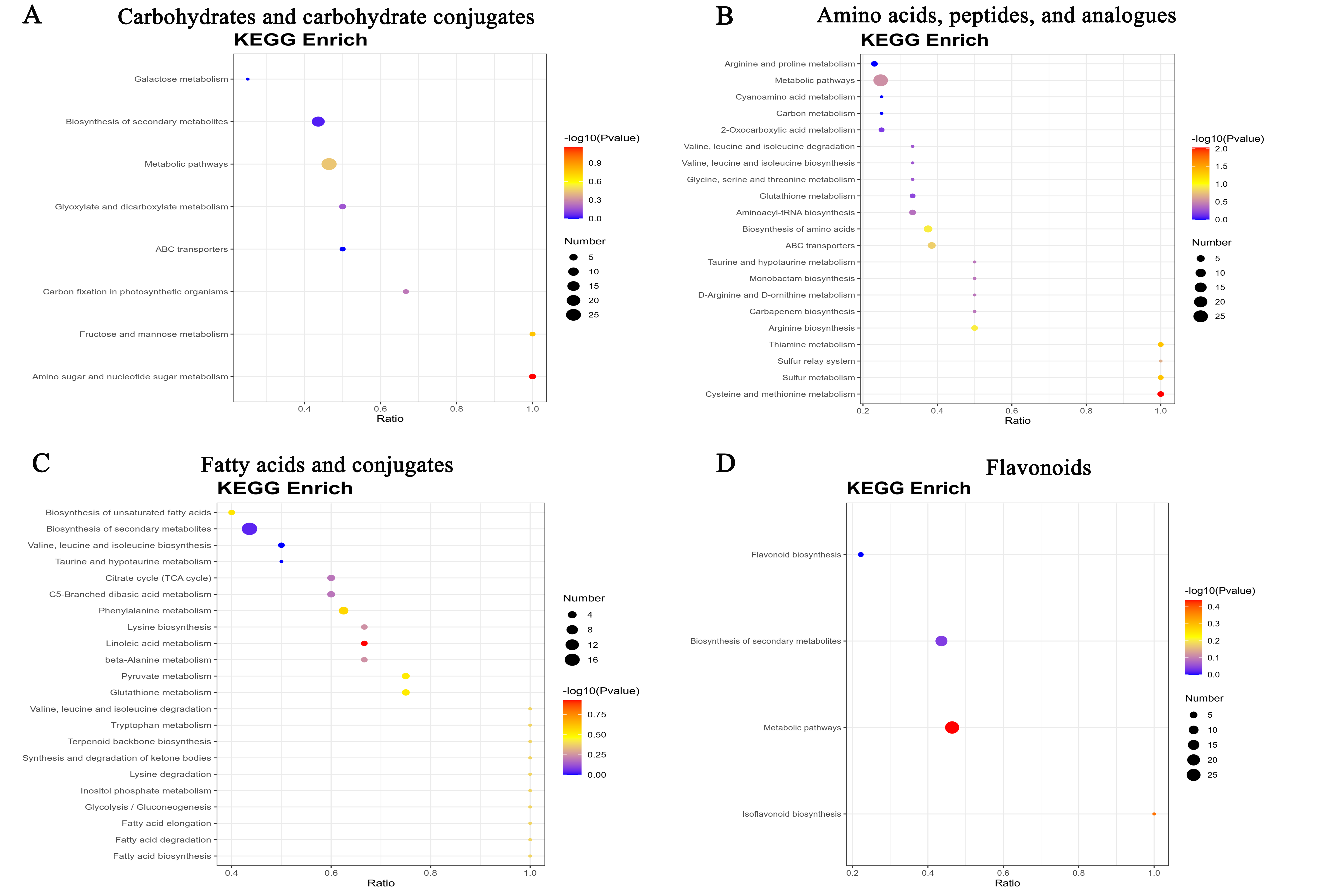

Supplement: Supplementary file 1 [file foods-13-00926-s001.zip › supplementary files/Fig. S3.tif]
